# Health shocks and households’ vulnerability to poverty in Nigeria: a quasi-experimental analysis

**DOI:** 10.1186/s13561-025-00660-5

**Published:** 2025-07-21

**Authors:** Paul Eze, Chimere O. Iheonu

**Affiliations:** 1 Department of Health Policy and Administration, Penn State University, 504 A Donald Ford Building, University Park, PA 16802 USA; 2 Department of Business Administration, African University of Science and Technology, Abuja, Nigeria

**Keywords:** Health shocks, Poverty, Vulnerability to poverty, Propensity score matching, Nigeria, I14, I15, I30, I31

## Abstract

Health shocks are a critical driver of poverty in low- and middle-income countries (LMICs). This study investigates the impact of health shocks on household poverty risk in Nigeria and evaluates the protective capacity of existing health insurance mechanisms, using data from the 2023/2024 General Household Survey (*n* = 4,779 households). We first estimated household vulnerability using Chaudhuri's (2003) Vulnerability as Expected Poverty framework, addressing heteroskedasticity in consumption estimation via feasible generalized least squares. We then modeled the probability of household vulnerability following health shocks using probit regression. Finally, we implemented propensity score matching to isolate the effect of health shocks on households’ vulnerability. Our results indicate that 56.1% and 59.2% of Nigerian households are vulnerable to poverty, using the international ($2.15/day) and national ($2.48/day) poverty lines, respectively. Vulnerable households are more prevalent in northern regions, rural areas, larger households, and poorer households. We found evidence that, based on the international poverty line, health shocks significantly increased the probability of household vulnerability (ATT = 0.053, SE = 0.023) compared to matched households without health shocks, with a slightly higher impact (ATT = 0.054, SE = 0.023) if using the national poverty line. Household size, dependency ratio, household head’s education and employment status, and residence were significant predictors of vulnerability. Despite their potential, health insurance, covering only 2.2% of households, did not offer adequate protection against health shocks. This study provides robust evidence that health shocks increase households' vulnerability to poverty and perpetuate existing poverty. Financial protection measures need to be considered in broader poverty reduction policies.

**Supplementary information:**

The online version contains supplementary material available at 10.1186/s13561-025-00660-5.

## Introduction

Health shocks – severe illnesses or injuries leading to substantial healthcare expenditures and/or loss of labor productivity – are a major driver of poverty, particularly in low- and middle-income countries (LMICs) [1]. These shocks often necessitate out-of-pocket (OOP) healthcare spending, forcing households to deplete savings, sell assets, reduce consumption, or withdraw children from school [2]. Concurrently, lost income due to illness and caregiving further exacerbates financial strain [3–5]. Beyond immediate financial depletion, health shocks often impede long-term investment in productive activities, perpetuating poverty [6, 7].

While poverty refers to the current state of individuals or households lacking the resources to meet basic needs, vulnerability to poverty is a forward-looking measure, assessing the risk of sliding into or remaining in poverty in the future [8–10]. Vulnerability to poverty captures the idea that today’s non-poor might not remain so in the future and today’s poor might not necessarily be poor tomorrow. Identifying vulnerable populations—both the currently poor and the at-risk non-poor—is crucial for targeted policy interventions [8, 9]. Addressing poverty and socioeconomic inequality is essential for improving social cohesion, stimulating economic growth, strengthening political stability, and ensuring equal access to opportunities. These goals are central to both global and national policy objectives, as well as Sustainable Development Goals (SDGs) 1 and 10 [11].

Nigeria, Africa’s most populous country with over 223 million people, faces severe poverty, with approximately 87 million people living below the international poverty line of US$2.15 per day – the second-largest poor population after India [12]. This constitutes a substantial portion of sub-Saharan Africa's (SSA) impoverished population [13]. Nigeria's health situation is significantly challenged by a high burden of infectious and non-communicable diseases (NCDs), exacerbated by widespread poverty and inadequate healthcare infrastructure [14]. Access to quality healthcare remains limited for many, with high OOP expenditures contributing to financial hardship and pushing vulnerable households deeper into poverty [15]. Consequently, key health indicators, such as crude, maternal, and child mortality rates, remain concerningly high, even compared to neighboring west African countries with lower income levels [14, 15].

Yet, to the best of our knowledge, there is no empirical evidence on the effect of health shocks on households’ vulnerability to poverty in Nigeria. In addition to Nigeria having the fastest-growing population of individuals living below the poverty line, this gap is significant for two main reasons. First, Nigeria's low health insurance coverage exposes households to catastrophic OOP expenditures from idiosyncratic (household-specific) health shocks. This vulnerability is further compounded by covariate (common) shocks like climate change, COVID-19, and conflicts [13]. Second, Nigeria’s large informal economy, employing up to 90% of the workforce, lacks income-protection during illness, such as paid sick leave [16].

This study investigates the impact of idiosyncratic health shocks on household vulnerability to poverty and evaluates the protective capacity of existing health insurance mechanisms. We aim to provide robust evidence on the extent to which health shocks exacerbate poverty, offering insights into their economic consequences in Nigeria and other LMICs. By identifying the most vulnerable subpopulations, our findings will inform the development of targeted social safety nets and health insurance policies for efficient resource allocation. Ultimately, our study highlights the significant economic impact of health shocks on poverty, reinforcing the importance of integrating health considerations into broader poverty reduction policies.

Our paper proceeds with an exploration of the conceptual framework of household vulnerability and coping strategies, followed by a review of recent empirical studies on these topics. We then describe the data and econometric methodology employed in our analysis. The subsequent section details the results of our study, and the paper concludes with a discussion of the major findings.

## Household vulnerability and coping strategies

We conceptualize the effect of health shocks on households’ vulnerability to poverty by considering a typical household influenced by external factors: physical, cultural, or political environment; and intrinsic factors: socioeconomic characteristics and resource endowments. Households utilize their resource endowments in various economic activities within their environmental contexts to generate income (through the production of goods and services), which is crucial for determining the household's vulnerability [17].

A household’s asset endowment includes capital and labor. Capital assets include natural (land), physical (livestock, houses), social (family network, community group), financial (bank savings, cash), and human (skills, knowledge, health). Labour endowment refers to the potential labor a household can provide for its own production or sell to external enterprises. Households aim to maximize consumption and leisure by utilizing their assets to produce value to society in the form of goods and services. They faces an income constraint, meaning total consumption, wages for hired labor, and rental payments cannot exceed the combined value of output, wage earnings, and earnings from rented land [17].

Households’ vulnerability to poverty is the ex-ante likelihood that a household will, if currently non-poor, fall below the poverty line (typically defined by a threshold of income or consumption), or if currently poor, remain in poverty [8, 9]. Vulnerability to poverty describes the probability of households becoming or remaining poor in the future due to floods, drought, health shocks, or job loss. While vulnerability reflects a forward-looking (ex-ante) measure of household welfare, poverty is an ex-post (observed) measure of household welfare [10]. Vulnerable households include currently poor households as well as non-poor households that would be impoverished given likely shocks [9]. Vulnerability is influenced by resource endowments and the capacity to mitigate risks from these shocks [8, 10].

Poverty and vulnerability are linked as the poor are most exposed to different risks and shocks, both idiosyncratic and covariates, due to insufficient resources to prevent them or mitigate their impact [18]. Without adequate coping mechanisms to address risks or mitigate the impact of shocks, these shocks can reduce households’ welfare by lowering capital formation and/or income [9, 10]. Coping mechanisms are efforts/actions households take to manage and mitigate the worst impact of risks and shocks [19]. Households experiencing health shocks commonly use coping mechanisms such as consuming less, depleting savings, receiving gifts and donations, borrowing, selling assets, and sending children to work. [4, 20, 21]. However, vulnerable households have less capacity to cope due to their limited resource endowments. In contrast, the wealthy non-poor typically do not need to adopt these measures, as they have sufficient resources to absorb the impact [19].

Coping strategies can be effective in the short term. Initial methods, such as selling livestock, relying on social support networks, utilizing social safety nets, and taking loans, are typically non-detrimental and help maintain essential productive resources [21]. However, some coping strategies can be detrimental, hindering households’ ability to recover. In such situations, households may be forced to sell productive assets, suffer health issues due to malnutrition, face difficulties accessing food and treatment, and prevent their children from attending school. In severe cases, individuals may experience family breakdowns or resort to crime and violence, leading to destitution [4, 21].

## Related literature

Research consistently shows that Nigerian households, like households in other LMICs, are highly vulnerable to poverty due to both idiosyncratic and covariate shocks including natural disasters such as droughts, floods, and pest outbreaks [22, 23], macroeconomic shocks like price fluctuations and inflation [24, 25], conflicts [22], and job loss [26, 27]. Although health shocks are the most common idiosyncratic shocks in Nigeria and other LMICs [28], there has been no empirical study on their impact on households'vulnerability to poverty in Nigeria. Studies in other SSA countries such as Ghana [29], Burkina Faso, Niger and Togo [6], Congo [30], South Africa [31], and elsewhere in India [4, 32, 33], China [34], and Pakistan [21], consistently show that health shocks exert a profound, significant, and often chronic effect on the economic vulnerability of exposed households.

Specifically, Novignon et al. (2012), utilizing data from Ghana's 2005/2006 Living Standards Survey, found that 56% of Ghanaian households were vulnerable to poverty, and health shocks worsen households’ vulnerability [29]. However, this foundational study did not examine the protective role of health insurance. Later, Ouadika (2020), utilizing data from the 2011 Congolese Household Survey, reported that 26.8% of households were vulnerable to poverty, with health shocks exacerbating this vulnerability [30]. Atake (2018) analysis of data from 3 SSA countries: Burkina Faso (Continuous Multi-Sector Survey, 2014), Niger (National Survey on Vulnerability to Food Insecurity of Households, 2014), and Togo (Core Welfare Indicators Questionnaire conducted, 2015) largely showed similar findings: 39.0% vulnerable households in Burkina Faso, 33.7% in Niger, and 69.0% in Togo, and health shocks worsened household vulnerability [6]. Importantly, this study showed that health insurance mitigated the vulnerability of households to poverty following health shocks. However, these studies relied on cross-sectional data and basic regression analysis, which limited their ability to fully account for selection bias. Morudu and Kollamparambil 2020 advanced the methodology in their South African study [31]. Using longitudinal data from three waves of the National Income Dynamics Study (2012–2017) and difference-in-difference analysis, they found that higher-income households were vulnerable to food expenditure shocks due to ill-health, though lower-income households were not. They also noted an insignificant protective effect of health insurance due to its very low penetration rate in the country. Further descriptive analyses from India [4, 32, 33], and Pakistan [21] also consistently show that health shocks exacerbate household vulnerability to poverty.

In related studies examining the impact of illness on household consumption in Ghana [35], Ethiopia [36], Nigeria [1], Indonesia [37], Pakistan [21], households often cope with the economic consequences of health shocks by decreasing non-food consumption. However, food expenditures are typically spared, and often increased, to address the health deficit and hasten recovery. This increase in food consumption is facilitated through coping strategies such as gifts, borrowing, selling assets, and own production [1, 35, 36]. Yet, coping strategies differ significantly by households’ socioeconomic status. Wealthy households with less resource constraints, if not insured, typically rely on their savings. In contrast, poorer households are more likely to resort to borrowing, selling land, cutting back on consumption, and working longer hours [4, 33]. Several studies suggest that health insurance, as a proactive measure to mitigate the impact of health shocks, protected insured households from sliding into poverty [6, 7, 29, 31, 32, 38]. In contrast, social protection programs such as disability assistance and unemployment benefits – when implemented in isolation, without concurrent health insurance coverage – had non-significant impact on the economic consequences of health shocks [35].

## Method

### Data sources

We utilized household-level data from the fifth wave (2023/2024) of the Nigeria General Household Survey (NGHS), a nationally representative survey conducted by Nigeria’s National Bureau of Statistics (NBS) in collaboration with the World Bank [39]. The NGHS provides comprehensive information on living conditions of civilian population across Nigeria's 36 states and the Federal Capital Territory (FCT), Abuja. This includes household demographics, education, health, labour, food and non-food expenses, non-farm enterprises, food security, housing conditions, household assets, and information and communication technology. Community-level data includes availability of infrastructure, transportation, and social utilities. The NGHS 2023/2024 employed a multi-stage sampling design to collect data from 4,779 households in 500 Enumeration Areas (EAs). Data were collected through face-to-face interviews conducted in two visits: post-planting (July – September 2023) and post-harvest (January – March 2024), with tracking of relocated households following each visit (October and November 2023 and April and May 2024). Data were gathered using computer-assisted interviews with live quality checks, then cleaned and reviewed for inconsistencies and extreme values.

### Study variables

In this study, health shocks are defined as any household member reporting episodes of illness within the four weeks preceding the survey. Previous studies have demonstrated the validity of self-reported episodes of illness as a measure for capturing major health shocks [6, 29, 30, 40]. We defined a'household'as a group of people who usually sleep in the same home and share meals [39].

We used expenditure, instead of income, as the welfare measure for computing our poverty estimates for three key reasons. First, this method aligns with the approach adopted by the Nigeria’s NBS in their poverty assessments. Second, it follows the conventional practice in countries with similar economic development levels to Nigeria. Lastly, and more importantly, the methodology for measuring expenditure in the NGHS-Panel surveys was “far more rigorous” than that for income [13]. To estimate the “expenditure aggregate” – our proxy for household welfare – we included seven essential components: (1) purchased food; (2) non-purchased food from own production or gifts; (3) meals consumed outside the home; (4) schooling and education costs; (5) expenditures for the healthcare of household members; (6) housing expenditures; and (7) expenditures on other non-food goods and services, including transport, fuel, electricity, household items, and clothing. We summed up estimates from these components to obtain household expenditure. This comprehensive approach ensures a holistic view of household welfare, reflecting not only monetary income but also the diverse ways in which households derive their sustenance and well-being [13]. We used two poverty lines to classify households as poor: the international extreme poverty line of US$2.15 per person per day in 2017 purchasing power parity (PPP), and the national poverty line of N137,430 per person per year (US$2.48 per person per day in 2017 PPP) from Nigeria’s NBS [39].

We included the following covariates, identified from related research [4, 6, 29, 30, 36]: household size (number of household members), dependency ratio (proportion of household members aged below 15 and above 64 years), health insurance, household wealth (computed as a principal component analysis (PCA) index of household assets [), ownership of house, housing condition, access to safe drinking water, access to improved sanitation, and setting (rural/urban) – Table 1. We categorized housing condition as either good or poor based on the primary construction materials of the floor, walls, and roof [41]. We included four variables that describe the household head: gender, age, educational attainment, and employment; and two interaction terms: health shock * health insurance, to estimate the protective effect of health insurance, and health shock * household wealth, to assess the mitigating effect of household wealth on households’ vulnerability to poverty.

**Table 1:** Sociodemographic characteristics, NGHS 2023/2024 (*n* = 4,779 households)

Sociodemographic characteristics	Number (%)
Household size, mean (SD)	6.7 (3.9) persons
Dependency ratio, mean (SD)	1.5 (1.4)
Household head gender, Female	1,088 (22.8%)
Household head age, mean (SD)	53.2 (15.0) years
Household head education, Primary or less	3,801 (79.5%)
Household head employment, Employed	4,242 (88.8%)
*Household wealth quintile*	
◦ ﻿Poorest	965 (20.2%)
◦ Poor	952 (19.9%)
◦ Middle	953 (19.9%)
◦ Rich	958 (20.1%)
◦ Richest	951 (19.9%)
Health insurance, Yes	105 (2.2%)
Residence, Rural	3,254 (68.1%)
Own house they dwell in, Yes	3,346 (70.0%)
House condition, Good	2,778 (58.1%)
Access to safe drinking water, Yes	3,232 (68.5%)
Access to adequate sanitation, Yes	2,792 (59.1%)
Food consumption per capita, median (IQR)	₦91,293 (₦45,476 – ₦172,073)
Non-food consumption per capita, median (IQR)	₦28,614 (₦12,903 – ₦58,163)
Total consumption per capita, median (IQR)	₦126,669 (₦65,902 – ₦234,907)
*Region/Geopolitical zones*	
◦ North Central	825 (17.3%)
◦ North East	816 (17.1%)
◦ North West	777 (16.3%)
◦ South East	798 (16.7%)
◦ South South	792 (16.5%)
◦ South West	771 (16.1%)

*Abbreviations*: *IQR* Interquartile range, *SD* Standard deviation

### Empirical methods

There are three main methods of assessing vulnerability: vulnerability as expected poverty (VEP), vulnerability as expected low utility (VEU), and vulnerability as uninsured exposure to risk (VER) [9]. VEP measures the probability that a household’s expected consumption expenditure will fall into poverty in the future [8]. VEU represents the difference in utility from a specific level of certainty-equivalent consumption, above which a household is not considered vulnerable [42]. Finally, VER assesses welfare loss after the fact, in the absence of an effective insurance mechanism, and evaluates the extent negative shocks result in welfare loss using panel data [42].

Vulnerability is determined by the expected average and variability of consumption, regardless of the estimation method. The average consumption is shaped by a range of individual, household, and community factors, while consumption variability is influenced by idiosyncratic and covariates shocks, as well as the households'capacity to employ coping strategies [42]. To evaluate the impact of health shocks on household vulnerability to poverty, we utilized the VEP approach, developed by Chaudhuri (2003), as it is easily understandable, explainable, forward-looking, and has been widely applied [42].

To empirically assess the impact of health shocks on households’ vulnerability to poverty in Nigeria, we followed the study of Novignon et al. (2012) by calculating households’ vulnerability to poverty. Accordingly, the likelihood that a household $h$ would go into consumption poverty at time $t+j$ is given as:

1 $${V}_{ht}=pr\left({lnC}_{n,t+j}<lnz\right)$$

Here, ${V}_{ht}$ indicates the vulnerability of a household $h$ at time $t$, ${lnC}_{n,t+j}$ represents the natural logarithm of consumption of household $h$ at time $t+j$. $z$ represents the poverty line of household consumption. The three-stage Feasible Generalized Least Squares (FGLS) suggested by Amemiya [43] is used in the construction of household vulnerability to poverty index. The initial procedure according to Koomson et al. [44] is to estimate the household consumption generating process. According to Atake [6], consumption is determined by a stochastic process, where:

2 $${lnC}_{ht}={\alpha y}_{h}+{u}_{h}$$

here: ${lnC}_{ht}$ is the natural logarithm of consumption per capita expenditure of household $h$, ${y}_{h}$ represents a vector of characteristics of the household and $\alpha$ is a vector of parameters to be estimated. ${u}_{h}$ is the error term. Koomson et al. [44] demonstrated that the squared estimated residual should be regressed on the same set of regressors as in Eq. (2) to account for variability in household consumption.

3 $${\widehat{\sigma }}_{ols,h}^{2}={y}_{h}\delta +{\vartheta }_{h}$$

The predicted values in Eq. (3) are obtained and used to transform Eq. (3), such that:

4 $$\frac{{\sigma }_{ols,h}^{2}}{{y}_{h}\widehat{\delta }}=\frac{{y}_{h}}{{y}_{h}\widehat{\delta }}+\frac{{\vartheta }_{h}}{{y}_{h}\widehat{\delta }}$$

Equation (4) when estimated by using the ordinary least squares (OLS) gives an asymptotically efficient FGLS estimate, ${\widehat{\delta }}_{FGLS}$. Furthermore, the standard deviation of Eq. (4) is derived in Eq. (5) and used to transform Eq. (4) to Eq. (6).

5 $${\widehat{\sigma }}_{e,h}=\sqrt{{y}_{h}{\widehat{\delta }}_{FGLS}}$$

6 $$\frac{{lnC}_{h}}{{\widehat{\sigma }}_{e,h}}=\left(\frac{{y}_{h}}{{\widehat{\sigma }}_{e,h}}\right)\alpha +\frac{{u}_{h}}{{\widehat{\sigma }}_{e,h}}$$

An estimation by OLS of Eq. (5) produces consistent and asymptotically efficient estimates of $\alpha$. Accordingly, estimating ${\alpha }_{FGLS}$ and ${\delta }_{FGLS}$ allows for a direct estimation of expected mean and variance for each household’s consumption as placed in Eqs. (7) and (8), respectively.

7 $$\widehat{E}\left[\left({lnC}_{h}|{y}_{h}\right)\right]={y}_{h}\widehat{\alpha }$$

8 $$\widehat{V}\left[\left({lnC}_{h}|{y}_{h}\right)\right]={\sigma }_{h}^{2}={y}_{h}\widehat{\delta }$$

Using estimates from Eq. (7) and (8), the probability of any given household $h$ with characteristics ${y}_{h}$ being vulnerable to poverty in the future can be estimated using Eq. (9) when the poverty line, vulnerability threshold and time horizon are known.

9 $${\widehat{V}}_{h}={\widehat{p}}_{r}\left({lnC}_{h,t+j}<lnz|{y}_{h}\right)=\varnothing \left(\frac{lnz-{y}_{h}\widehat{\alpha }}{\sqrt{{y}_{h}\widehat{\delta }}}\right)$$

Following extant literature, we employ a vulnerability to poverty threshold of 0.5 [29, 43, 45]. The choice of 0.5 vulnerability threshold is validated based on the following reasons, (1) it is intuitively reasonable to suggest that a household with a 50% likelihood of falling into poverty in the next period can be considered vulnerable to poverty, and (2) when a household’s current consumption level matches the poverty line and it experiences a shock with a zero mean, its vulnerability to poverty one period ahead is 0.5. As the time horizon approaches zero, the notions of being currently poor and being vulnerable to poverty converge.

To examine the relationship between health shocks and households’ vulnerability to poverty, we employed two approaches. In the first approach, we modelled the probability of a household being vulnerable to poverty using probit regression. Specifically, the probability of vulnerability, denoted as $\text{Pr}\left({V}_{t}{P}_{i}=1|{X}_{i}\right)$, is a function of various household characteristics and health shock exposure:

10 $$\text{Pr}\left({VtP}_{i} =1 \right| {X}_{i})=\Phi ( {\beta }_{0} + {\beta }_{1}{Healthshock}_{i} + {\beta }{\prime}{X}_{i} +{\varepsilon }_{i} )$$

Here: $$\text{Pr}\left({VtP}_{i} =1 \right| {X}_{i})$$ is the probability that household *i* is vulnerable to poverty, measured using two distinct poverty thresholds: $2.15/day and $2.48/day. $$\Phi$$ is the cumulative distribution function of the standard normal distribution. $${\beta }_{0}$$ is the intercept. $${\beta }_{1}$$ is the coefficient for the HealthShock_*i*_, $${\beta }{\prime}$$ is the coefficient for the covariates. $${X}_{i}$$ is a vector of covariates for household *i*, including household size, dependency ratio, gender, age, education, and employment of the household head, household wealth, health insurance, home ownership, housing condition, residence, and the interaction terms: *HealthShock*_*i*_ × *HouseholdWealth*_*i*_, and *HealthShock*_*i*_ × *HealthInsurance*_*i*_. $${\varepsilon }_{i}$$ is the error term representing all the unobserved factors that may influence the dependent variable (vulnerability to poverty) but are not included in the model. $$i$$ represents the cross-sectional index. We conducted 10,000 bootstrap replications to obtain robust standard errors.

In the second approach, we estimated the causal effect of idiosyncratic health shocks on households’ vulnerability to poverty using propensity score matching (PSM). Given the observational nature of our data, households were not randomly assigned to health shock exposure, potentially introducing selection bias. To address this, we aimed to balance the distributions of observable confounding factors between households experiencing health shocks (the"treatment"group) and those without (the"control"group). We estimated propensity scores—the conditional probability of a household's health shock exposure given a vector of observed covariates—using a logistic regression model. The propensity score, denoted as:

11 $$e\left(X\right)=\text{Pr}(T=1\left|X\right.)$$

represents the probability of receiving treatment (health shock) given a set of observed covariates *X*, where *T* is the treatment indicator (1 for treatment, 0 for control). This approach relies on the conditional independence assumption (CIA), which posits that, conditional on the propensity score, potential outcomes are independent of treatment assignment [46, 47]. We matched households in the treatment group to those in the control group based on the absolute difference in their estimated propensity scores:

12 $${VS}_{\text{ij}}=\left|{PS}_{\text{i}}-{PS}_{\text{j}}\right|$$

where *PS*_*i*_ and *PS*_*j*_ represent the propensity scores for households in the treatment and control groups, respectively. This method effectively creates a counterfactual control group that is statistically similar to the treated group in terms of observed covariates, thereby mitigating selection bias [48, 49].

To enhance the precision of our matching, we made a few methodological refinements. First, instead of using categorical variables for local government area (county), state, and region, we incorporated three continuous, aggregate community-level indices: socioeconomic infrastructure, utilities infrastructure, and transportation infrastructure. The socioeconomic infrastructure index is a PCA index of availability of schools, health facilities, markets, banks, and microfinance institutions in the community. The utilities infrastructure index is a PCA index of availability of cell phone distributors (as a proxy for the availability of mobile telecommunication), post offices, police stations, fire stations, community centres, and religious centres in the community. The transportation infrastructure index is a PCA index of presence of bus stops and paved access roads. These indices provide a more nuanced representation of the community context. Second, we utilized the raw PCA summary scores of the household wealth index, rather than categorical household wealth quintiles, to capture finer gradations in household wealth. Finally, we implemented one-to-one nearest neighbour matching without replacement, imposing a 0.01 calliper restriction to ensure that matched pairs were sufficiently similar in their propensity scores. Following Rubin 2001 [50], we evaluated covariate balance using standardized percentage bias, aiming to reduce biases below 5%.

We then estimated the average treatment effect on the treated (ATT), defined as:

13 $$\text{ATT}=E\left({Y}_{1}\left|D=1\right.\right)-E\left({Y}_{0}\left|D=1\right.\right)$$

where *D* is the treatment indicator (health shock), *Y*_1_ is the outcome if treated, and *Y*_0_ is the outcome if not treated. The ATT represents the difference between the average likelihood of vulnerability in health shock-exposed households and the average likelihood of vulnerability in the control group.

Given the potential for heterogeneity in factors influencing treatment assignment across subgroups, we computed separate propensity scores for our subgroup analyses. This subgroup-specific approach ensures covariate balance tailored to the unique characteristics of each group, enhancing the internal validity of subgroup-specific ATT estimates. For both the main (full sample) and subgroup analyses, we reported both Abadie Imbens (AI) and bootstrap (1,000 replications) robust standard errors (SE) [51]. All analyses were conducted in Stata 18.5 SE using the *psmatch2* Stata ado command for PSM [52].

## Results

Our study sample consisted of 4,779 households, nearly equally selected from the six geopolitical zones – Table 1. On average, households comprised 6.7 persons, with a dependency ratio of 1.5. Most households were male headed (77.2%), had an employed household head (88.8%), resided in rural areas (68.1%), and were uninsured (97.8%). The median annual per capita expenditure for food, non-food, and total expenses were ₦91,293 ($601), ₦28,614 ($189), and ₦126,669 ($834), respectively. Approximately 20.4% of households in our study sample experienced health shocks in the four weeks preceding the survey.

The proportion of vulnerable households, using the international poverty line and national poverty line, was 56.1% and 59.2%, respectively – Table 2. Across both poverty lines, current poverty status was significantly associated with vulnerability to poverty (*p* < 0.001), indicating that most vulnerable households are currently poor. This national prevalence conceals regional and sociodemographic disparities, with the highest prevalence observed in the North West region (67.0% for the international poverty line and 71.7% for the national poverty line) and the lowest in the North Central region (40.6% for the international poverty line and 46.0% for the national poverty line) – Table 3. Across both poverty lines, the prevalence of vulnerability to poverty was higher among rural households, female-headed households, larger households, and the poorest households.

**Table 2:** Poor and vulnerable households, NGHS 2023/2024 (incorporating sample weights)

		**Vulnerability to poverty**	
		**Not vulnerable**	**Vulnerable**
(**A**).International poverty line: $2.15 per person per day, based on 2017 PPP			
Poverty rate	Non-poor	Not poor and not vulnerable 33.2%	Not poor but vulnerable 25.9%
	Poor	Currently poor but not vulnerable 10.7%	Currently poor and vulnerable 30.2%
(**B**). National (Nigeria) poverty line: $2.48 per person per day, based on 2017 PPP			
Poverty rate	Non-poor	Not poor and not vulnerable 29.9%	Not poor but vulnerable 22.9%
	Poor	Currently poor but not vulnerable10.9%	Currently poor and vulnerable36.3%

*P*-value for Pearson’s Chi-square test = < 0.001

**Table 3:** Vulnerability to poverty profile for various population characteristics, NGHS 2023/2024

	Population share **	**International poverty line**		**National (Nigeria) poverty line**	
		**Mean vulnerability**	**Vulnerability to population ratio**	**Mean vulnerability**	**Vulnerability to population ratio**
Total (National)	100%	56.1%	100%	59.2%	100%
Regions					
◦ North Central	14.3%	40.6%	10.3%	46.0%	11.1%
◦ North East	14.5%	75.6%	19.6%	81.4%	20.0%
◦ North West	27.2%	67.0%	32.5%	71.7%	39.2%
◦ South East	10.8%	52.2%	10.1%	53.0%	9.7%
◦ South South	14.6%	46.6%	12.2%	47.3%	11.7%
◦ South West	18.6%	46.4%	15.3%	46.8%	14.7%
Setting					
◦ Rural	65.6%	63.9%	74.8%	68.1%	75.5%
◦ Urban	34.4%	41.0%	25.2%	42.2%	24.5%
Gender of household head					
◦ Female	15.5%	50.9%	14.1%	54.4%	15.5%
◦ Male	84.5%	56.9%	85.9%	60.1%	84.5%
Household size					
◦ ≤ 6 persons	57.9%	37.4%	24.1%	38.8%	36.1%
◦ > 6 persons	42.1%	66.6%	75.9%	70.8%	63.9%
Household wealth quintile					
◦ Poorest	19.0%	79.8%	27.0%	86.8%	27.8%
◦ Poor	19.1%	66.2%	22.6%	71.3%	23.1%
◦ Middle	21.5%	62.1%	23.9%	65.6%	23.9%
◦ Rich	20.0%	50.1%	17.9%	50.5%	17.0%
◦ Richest	20.4%	23.6%	8.6%	23.8%	8.2%

** Weighted using sampling weights

The probit model results reveal several significant factors associated with households’ vulnerability – Table 4. Experiencing a health shock was positively and significantly associated with an increased probability of household vulnerability using the international poverty line: coefficient = 0.149, SE = 0.0634, *p* = 0.019, marginal effect = 0.044 (see columns 2 to 5, Table 4) and similarly significant using the national poverty line: coefficient = 0.153, SE = 0.0672, *p* = 0.023, marginal effect = 0.043 (see columns 6 to 9, Table 4). Consistent across both poverty lines, larger household size, higher dependency ratio, and rural residence significantly increased the likelihood of household vulnerability to poverty. Conversely, higher educational attainment and employment of the household head were associated with a significantly lower probability of vulnerability to poverty. Both interaction terms: ‘health shock * household wealth’ and ‘health shock * health insurance’ were associated with a lower probably of household’s vulnerability, although these were not statistically significant.

**Table 4:** Probit regression for household vulnerability to poverty, NGHS 2023/2024

	**International poverty line**				**National (Nigeria) poverty line**			
**Variables**	**Coefficients**	**Bootstrap SE**	***p*-value**	**Marginal effects**	**Coefficients**	**Bootstrap SE**	***p*-value**	**Marginal effects**
Exposure variable								
Health shock	0.149	0.063	0.019**	0.044	0.153	0.067	0.023**	0.043
Household characteristics								
Household size	0.139	0.035	0.000***	0.041	0.129	0.029	0.000***	0.037
Square of household size	0.001	0.002	0.796	0.000	0.002	0.002	0.315	0.001
Dependency ratio	0.130	0.022	0.000***	0.039	0.131	0.022	0.000***	0.037
Gender of household head	−0.089	0.057	0.117	−0.026	−0.078	0.057	0.170	−0.022
Age of household head	−0.010	0.009	0.297	−0.003	−0.009	0.009	0.364	−0.003
Square of age of household head	−0.000	0.000	0.988	−0.000	−0.000	0.000	0.821	−0.000
Educ. of household head	−0.386	0.082	0.000***	−0.113	−0.364	0.083	0.000***	−0.102
Employment of household head	−0.788	0.089	0.000***	−0.232	−0.796	0.097	0.000***	−0.223
Household wealth quintile								
◦ Poorest	1.226	0.119	0.000***	0.373	1.388	0.122	0.000***	0.408
◦ Poor	0.774	0.104	0.000***	0.236	0.873	0.106	0.000***	0.262
◦ Middle	0.565	0.096	0.000***	0.170	0.624	0.097	0.000***	0.186
◦ Rich	0.394	0.087	0.000***	0.117	0.399	0.088	0.000***	0.117
Health insurance	0.108	0.209	0.605	0.032	0.106	0.210	0.612	0.030
Own house they dwell in	−0.032	0.068	0.644	−0.009	−0.069	0.067	0.305	−0.019
Housing condition	−0.020	0.063	0.752	−0.006	−0.071	0.063	0.256	−0.020
Residence	0.191	0.087	0.029**	0.057	0.165	0.087	0.062*	0.047
Interaction terms								
Health shock ## Household wealth	−0.030	0.072	0.676	−0.009	−0.024	0.072	0.740	−0.007
Health shock ## Health insurance	−0.210	0.242	0.384	−0.062	−0.233	0.243	0.336	−0.066
Number of observations	4,743				4,743			
McFadden’s R2	0.249				0.279			
Percentage of correctly classified	74.9%				76.3%			

^*^ *p* < 0.10, ** *p* < 0.05, *** *p* < 0.01

**Fig. 1:**
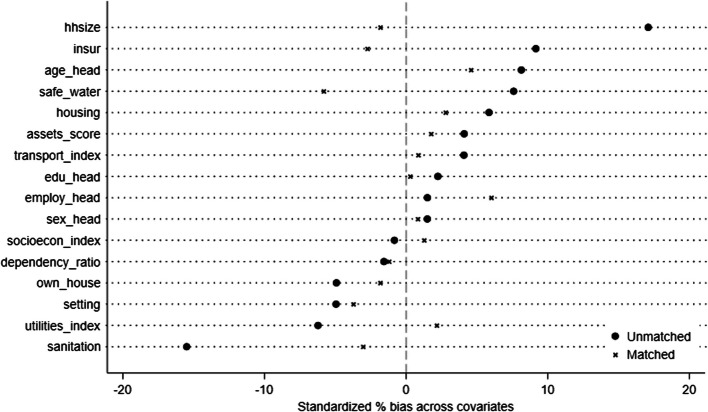
Love plot of standardized percentage bias before and after propensity score matching

**Fig. 2:**
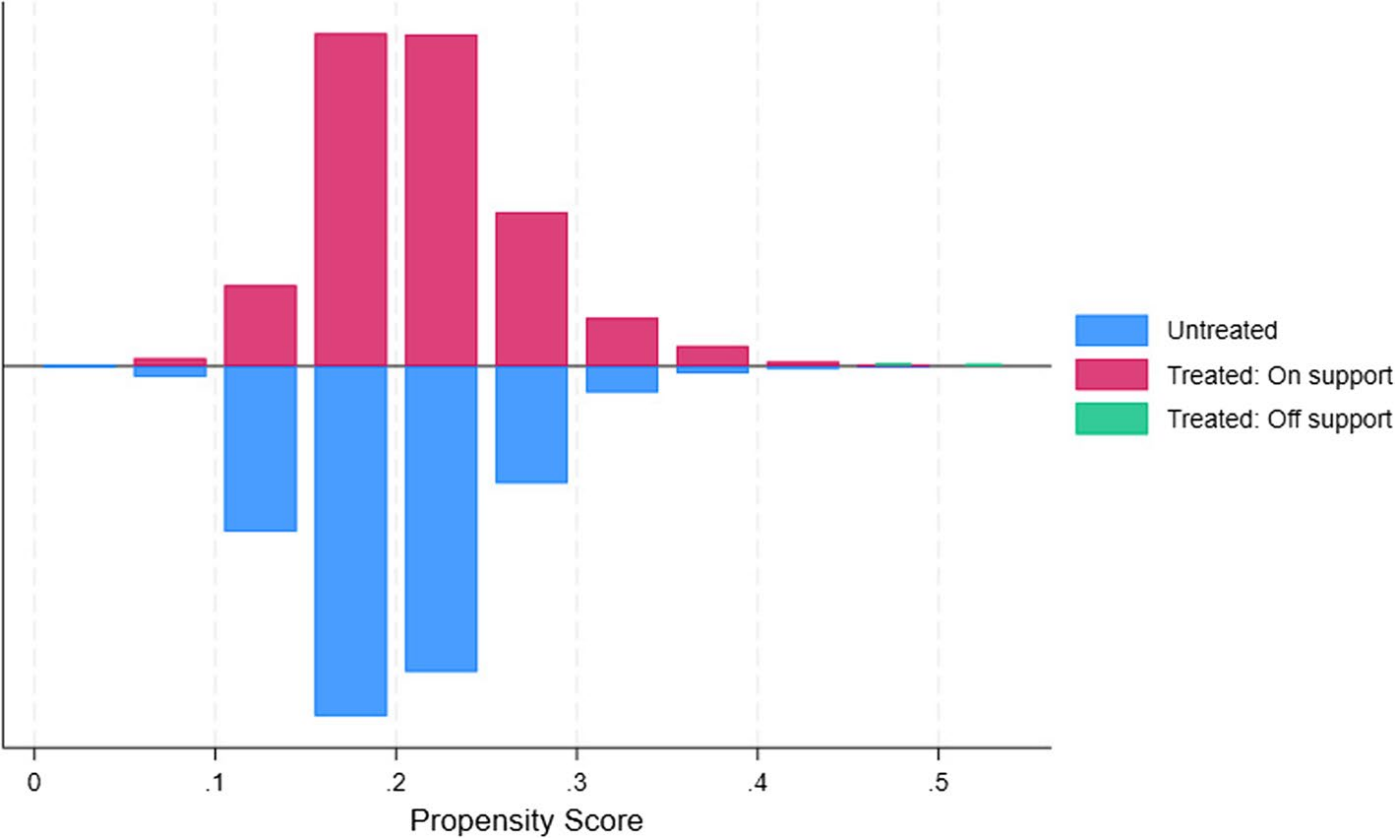
Matching graph of the propensity score before and after propensity score matching

**Fig. 3:**
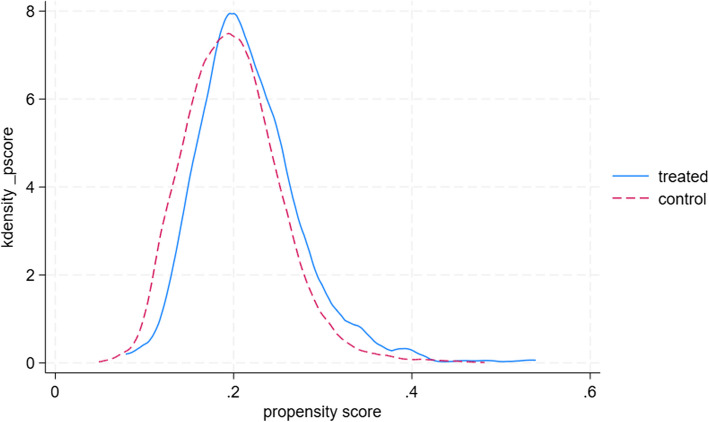
Covariate balance with propensity scores

Table 5 and Fig. 1 presents the balance diagnostics for the covariates before (unmatched) and after PSM (matched). Prior to matching, notable differences existed between treated and control households. For instance, treated households had a larger average household size (7.45 vs. 6.74, % Bias = 17.1) and a higher percentage of households with access to safe water (35% vs. 32%, % Bias = 7.6). The mean percentage bias across all covariates before matching was 6.0%, with a median bias of 4.9% and a Rubin's B of 34.0. Following PSM, the balance across these covariates improved substantially. The mean household size became similar in both groups (7.41 vs. 7.49, % Bias = −1.8), and the percentage bias for access to safe water reduced to −5.8%. Overall, after matching, the mean percentage bias decreased to 2.6%, the median bias to 2.2%, and Rubin's B reduced to 11.1, indicating a considerable reduction in the imbalance of observed covariates between the treated and control groups [49]. Rubin's R remained close to 1 (1.09), suggesting that the variance ratio of the propensity scores between the treated and control groups was acceptable after matching. The common support assumption was satisfied, as shown in Figs. 2 and 3.

**Table 5:** Covariate balance before and after matching, NGHS 2023/2024

Variable	Unmatched/Matched	Exposed households	Control households	% Bias*
Household size, mean	U	7.45	6.74	17.1
	M	7.41	7.49	−1.8
Dependency ratio, mean	U	1.29	1.31	−1.6
	M	1.29	1.30	−1.3
Gender of household head, %	U	0.23	0.22	1.5
	M	0.23	0.23	0.8
Age of household head, mean	U	54.41	53.20	8.1
	M	54.37	53.69	4.6
Education of household head, %	U	0.22	0.21	2.2
	M	0.22	0.21	0.6
Employment of household head, %	U	0.88	0.88	1.5
	M	0.88	0.86	6.0
Household wealth index, mean	U	−0.06	−0.16	4.1
	M	−0.06	−0.12	2.2
Health insurance, %	U	0.99	0.98	9.2
	M	0.99	0.99	−2.7
Own house, %	U	0.25	0.27	−4.9
	M	0.25	0.26	−1.8
Housing condition, %	U	0.60	0.57	5.8
	M	0.60	0.58	3.0
Access to safe water, %	U	0.35	0.32	7.6
	M	0.35	0.38	−5.8
Adequate sanitation, %	U	0.36	0.44	−15.5
	M	0.37	0.38	−2.8
Residence, %	U	0.68	0.71	−4.9
	M	0.68	0.70	−4.0
Socioeconomic infrastructure index, mean	U	−0.06	−0.04	−0.8
	M	−0.06	−0.09	1.3
Utilities infrastructure index, mean	U	−0.07	0.02	−6.2
	M	−0.07	−0.10	2.2
Transportation infrastructure index, mean	U	0.02	−0.03	4.1
	M	0.02	0.01	1.0
Sample	Mean Bias	Median Bias	Rubin’s B	Rubin’s R
Unmatched	6.0	4.9	34.0	1.02
Matched	2.6	2.2	11.1	1.09

^*^This column presents the standardized percentage bias, both before and after matching, which quantifies the difference in mean values between the treated and control groups as a percentage of the average standard deviation [52]

After reducing the mean standardized bias for the covariates, we assessed the ATT to examine whether health shock exposure had a causal impact on households’ vulnerability – Table 6. Using the international poverty line, households that experienced a health shock had a 5.3 percentage points higher probability of being vulnerable (ATT = 0.053, AI robust SE = 0.0233, 95% CI: 0.007, 0.098, *p* = 0.024). Similar results were observed when employing the national poverty line, with health-shocked households exhibiting a 5.4 percentage point higher probability of poverty vulnerability (ATT = 0.054, AI robust SE = 0.0231, 95% CI: 0.008, 0.099, *p* = 0.020). Bootstrap analysis with 1,000 replications yielded smaller SE and narrower confidence intervals. These findings suggest a statistically significant and positive impact of health shocks on households'vulnerability, irrespective of the poverty line used.

**Table 6:** ATT estimates of health shocks on households’ vulnerability, NGHS 2023/2024

Poverty line	Effect estimates	AI robust SE	AI 95% Confidence interval	Bootstrap SE*	Bootstrap 95% Confidence interval*	*p*-value
International poverty line	0.053	0.023	0.007, 0.098	0.001	0.050, 0.055	0.024
National (Nigeria) poverty line	0.054	0.023	0.008, 0.099	0.001	0.051, 0.056	0.020

^*^Bootstrap standard errors and 95% confidence interval were estimated by bootstrapping with 1,000 replications

### Robustness tests

There are three primary concerns regarding the robustness of causal estimates derived from PSM, such as ours. First, the potential presence of hidden bias due to unobserved confounders that affects both the treatment assignment (health shock) and outcome (households’ vulnerability to poverty) but were not included in our PSM model. Such hidden bias could come from latent household health. Second, a violation of our main identifying assumption – the CIA – which is fundamental to the validity of our ATT estimates. Third, the potential model dependence of estimated treatment effects to the specific matching algorithm employed.

To address the first concern of hidden bias, we conducted a Rosenbaum bounds sensitivity analysis [53]. This test assesses how strong an unobserved confounder would need to be to alter our conclusions about the ATT estimates. We examined increasing levels of gamma (Γ), representing the odds ratio of differential treatment assignment due to an unobserved covariate, from 1.0 (no hidden bias) to 2.0. Table 7 presents the results, where *p*-values for the ATT are evaluated at each gamma level, reflecting the potential endogeneity of treatment assignment. The results indicate that even with increasing levels of potential hidden bias, the lower bound of the significance level (Sig-) remains below the conventional 0.05 threshold until gamma reaches 1.2 (additional analysis, not shown here, suggests this holds until approximately gamma = 1.29). At gamma = 1.3, however, the lower bound of the 95% confidence interval (CI-) crosses zero, suggesting that the ATT estimate is no longer statistically significant if an unobserved confounder increases the odds of treatment by a factor of 1.3. This suggests that our main ATT estimates are relatively robust to moderate levels of unobserved confounding. However, it is plausible that unobserved confounders, such as latent household health, exerting such a strong influence (gamma ≥ 1.3) will likely correlate with observable indicators already captured in our treatment variable.

**Table 7:** Rosenbaum bounds robustness tests for the ATT estimates, NGHS 2023/2024

**Gamma**	**Sig+**	**Sig-**	*t-hat+*	*t-*hat-	**CI+**	**CI-**
International poverty line						
1	0.010	0.010	−4.1 e-07	−4.1 e-07	−4.1 e-07	−4.1 e-07
1.1	0.085	0.001	−4.1 e-07	−4.1 e-07	−4.1 e-07	−4.1 e-07
1.2	0.309	1.4 e-05	−4.1 e-07	−4.1 e-07	−4.1 e-07	−4.1 e-07
1.3	0.620	2.8 e-07	−4.1 e-07	−4.1 e-07	−4.1 e-07	4.1 e-07
1.2	0.853	3.9 e-09	−4.1 e-07	−4.1 e-07	−4.1 e-07	4.1 e-07
1.5	0.959	4.1 e-11	−4.1 e-07	−4.1 e-07	−4.1 e-07	4.1 e-07
National (Nigeria) poverty line						
1	0.011	0.011	−4.1 e-07	−4.1 e-07	−4.1 e-07	−4.1 e-07
1.1	0.093	0.001	−4.1 e-07	−4.1 e-07	−4.1 e-07	−4.1 e-07
1.2	0.331	1.5 e-05	−4.1 e-07	−4.1 e-07	−4.1 e-07	−4.1 e-07
1.3	0.647	2.7 e-07	−4.1 e-07	−4.1 e-07	−4.1 e-07	4.1 e-07
1.4	0.871	3.5 e-09	−4.1 e-07	−4.1 e-07	−4.1 e-07	4.1 e-07
1.5	0.967	3.5 e-11	−4.1 e-07	−4.1 e-07	−4.1e-07	

Next, to evaluate the robustness of our ATT estimates to violations of the CIA, we employed a simulation method developed by Ichino et al. [54]. This method simulates a potential binary unobserved confounder by drawing on the distribution of observed variables in our data, effectively estimating the potential bias from unobservables like latent household health by mimicking the influence of relevant observed factors such as household head educational attainment and employment. We also simulated varying distributions of this relevant unobserved confounder that could drive the estimated treatment effect to zero. This approach allows us use different assumptions about the distribution of this relevant unobserved confounder to assess the robustness of our ATT estimates [54]. Table 8 compares our baseline ATT estimates (see Table 6) with the average simulated ATT obtained from 500 repetitions under varying distribution of this potential unobserved confounding. The ATT estimates derived using this simulation method is slightly larger than our baseline ATT estimates (see Table 6). However, the results demonstrate that our ATT estimates are robust to the presence and varying distribution of unobserved confounding (Table 8).

**Table 8:** Sensitivity analyses of ATT estimates to unobserved confounder using the *sensatt* simulation method

	**p11**	**p10**	**p01**	**p00**	**d**	**s**	**Outcome effect**	**Selection effect**	**ATT (SE)**
*International poverty line *									
No confounder	0.00	0.00	0.00	0.00					0.054 (0.026)
Confounder like household head education	0.17	0.25	0.19	0.22	0.20	0.20	0.843	1.007	0.054 (0.026)
Confounder like household head employment	0.84	0.94	0.81	0.95	0.89	0.88	0.211	1.034	0.054 (0.026)
Latent household health – I	0.20	0.15	0.15	0.10	0.18	0.12	1.597	1.521	0.054 (0.026)
Latent household health – II	0.50	0.20	0.20	0.12	0.36	0.16	1.825	2.952	0.054 (0.026)
Latent household health – III	1.00	0.25	0.25	0.15	0.64	0.20	1.905	7.258	0.054 (0.026)
Latent household health – IV	1.00	0.50	0.50	0.20	0.76	0.35	4.023	6.104	0.054 (0.026)
*National (Nigeria) poverty line *									
No confounder	0.00	0.00	0.00	0.00					0.055 (0.026)
Confounder like household head education	0.18	0.24	0.20	0.21	0.20	0.20	0.893	1.003	0.055 (0.026)
Confounder like household head employment	0.84	0.94	0.81	0.96	0.89	0.88	0.197	1.038	0.055 (0.026)
Latent household health – I	0.20	0.15	0.15	0.10	0.18	0.13	1.602	1.498	0.055 (0.026)
Latent household health – II	0.50	0.20	0.20	0.12	0.36	0.16	1.846	2.994	0.055 (0.026)
Latent household health – III	1.00	0.25	0.25	0.15	0.66	0.20	1.928	7.589	0.055 (0.026)
Latent household health – IV	1.00	0.50	0.50	0.20	0.77	0.35	4.027	6.120	0.055 (0.026)

Lastly, to address concerns about the sensitivity of our findings to the specific matching algorithm, we utilized alternative PSM methods. The ATT estimates derived from these different algorithms were consistently statistically significant and of similar magnitude to our main findings – Table 9. This consistency across various matching algorithms strengthens confidence in our findings, suggesting a robust causal impact of health shocks on households’ vulnerability. As Caliendo and Kopeinig [49] noted, different matching methods should converge to similar ATT estimates asymptotically, as it is the case in our analysis of the NGHS 2023/2024 data. Furthermore, our ATT estimates remained robust even when the propensity scores were computed using a probit model instead of the primary logit specification [55].

**Table 9:** Robustness of the ATT estimates using alternative matching algorithms

	**International poverty line**			**National (Nigeria) poverty line**		
**Alternative methods**	**ATT estimates**	**AI robust SE**	**Bootstrap SE**	**ATT estimates**	**AI robust SE**	**Bootstrap SE**
1-to-2 nearest neighbour matching	0.053	0.0233**	0.0010**	0.054	0.0231**	0.0011**
1-to-3 nearest neighbour matching	0.053	0.0233**	0.0009**	0.054	0.0231**	0.0009**
1-to-5 nearest neighbour matching	0.053	0.0233**	0.0008**	0.054	0.0231**	0.0008**
Caliper/radius 0.02 matching	0.053	0.0233**	0.0012**	0.054	0.0231**	0.0012**
Caliper/radius 0.05 matching	0.053	0.0233**	0.0012**	0.054	0.0231**	0.0012**
Caliper/radius 0.10 matching	0.053	0.0233**	0.0013**	0.054	0.0231**	0.0012**
Kernel matching	0.053	0.0233**	0.0005**	0.054	0.0231**	0.0005**
Propensity scores using probit model	0.053	0.0233**	0.0012**	0.054	0.0231**	0.0012**

^*^ *p* < 0.10, ** *p* < 0.05, *** *p* < 0.01

### Subgroup analysis

Our exploration of NGHS 2023/2024 data to assess the influence of health shocks on households'vulnerability across relevant sociodemographic groups was only partly successful. The small sample size within the exposed and control groups of relevant groups such as insured vs. uninsured households, male-headed vs. female-headed households, and health shocks involving the household head vs. those involving other members [56], hindered our ability to achieve covariate balance necessary for valid ATT estimates. For example, there are only 12 households in the treated arm in the insured vs. uninsured households’ subgroup analysis. Thus, we only successfully examined the influence of health shocks across residential areas. Among rural households, experiencing a health shock was associated with a statistically significant increase in the probability of being vulnerable. Specifically, using the international poverty line, rural households experiencing a health shock had a 6.9 percentage point higher probability of vulnerability – Table 10 (see Tables 2 and 3, Figures A5–A10 for results of covariate balance). However, the ATT estimates for urban households were not statistically significant for both poverty lines.

**Table 10:** ATT estimates of health shocks on households’ vulnerability across residential areas, NGHS 2023/2024

	**International poverty line**			**National (Nigeria) poverty line**		
	**ATT estimates**	**AI Robust SE**	**Bootstrap SE**	**ATT estimates**	**AI Robust SE**	**Bootstrap SE**
Residence						
◦ Rural households	0.069	0.0286**	0.0017**	0.067	0.0290**	0.0016**
◦ Urban households	−0.004	0.0367	0.0032	0.011	0.0372	0.0034

* *p* < 0.10, ** *p* < 0.05, *** *p* < 0.01

## Discussion

This study examined the effects of idiosyncratic health shocks on households’ vulnerability to poverty in a representative sample of Nigerian households. To the best of our knowledge, this is the first study to use PSM to explore the implications of health shocks on future household welfare. Using nationally representative data and PSM, we provide robust evidence of the adverse impact of health shocks on household future welfare, specifically their vulnerability to poverty. Irrespective of the poverty threshold applied, our probit regression models consistently show that health shocks significantly increase households'risk of future poverty or perpetuate existing poverty. We also observe that health insurance, in the event of health shocks, substantially mitigates, albeit statistically insignificant, the impoverishing effects of these shocks. Similarly, household wealth provides a mitigating effect, though considerably smaller in magnitude and also statistically insignificant. Our PSM analyses robustly demonstrates that health shocks significantly increase household poverty risks. The health shock-induced risks further adds to the existing poverty-exacerbating risks of aggregate climate and economic shocks [21, 28].

Our finding that health shocks significantly increase households’ vulnerability to poverty is consistent with prior research that examined the effects of health shocks on household consumption [1, 4, 36, 57], and vulnerability to poverty [6, 29, 30]. Specifically, our results are most directly comparable to those of Ouadika [30], Atake [6], and Novignon et al. [29] which utilized similar nationally representative datasets, outcome measures, and focused on SSA countries. Although these studies did not employ quasi-experimental methods to estimate causal effects, they report broadly similar associations. Specifically, consistent with our findings, they identify large household size, lower socioeconomic status, and low educational attainment of the household head as significant predictors of household vulnerability. However, a few differences emerge: Atake [6] found a significant mitigating effect of health insurance (we observed a similar, though statistically insignificant, trend); Novignon et al. [29] reported significantly lower vulnerability in rural households and no significant association between poverty and vulnerability; and Ouadika [30] and Atake [6]found female-headed households to be more vulnerable (we observed the opposite, though not statistically significant).

Our failure to observe a statistically significant protective effect of health insurance on household vulnerability to health shocks is consistent with findings from India [4] and a large literature review by Alam & Mahal, 2014 [2]. This contrasts with more recent studies from Burkina Faso, Niger, and Togo [6], Ghana [7], and Vietnam [38] which show significant protective effects. We suspect that the lack of statistical significance in our study is due to insufficient statistical power to detect the protective effect, likely due to the extremely low insurance coverage rate in our sample (2.2%). This low coverage mirrors the 3.7% coverage observed in India [4], potentially explaining the similar findings. Taken together, these results suggest that the capacity of health insurance to effectively buffer households against health shocks may be contingent upon achieving a substantial level of population coverage, as evidenced in higher-coverage settings [7, 58]. Policy-wise, this suggests the need to expand health insurance coverage in Nigeria, and in similar contexts, to effectively mitigate households’ vulnerability.

Beyond providing robust estimates, our analysis reveal a significant disparity in the impact of health shocks on households’ vulnerability based on residence. Specifically, health shocks had a more pronounced impoverishing effect on rural households experience compared to their urban counterparts, consistent with findings in neighbouring Burkina Faso, Niger, and Togo [6], and elsewhere in China [34], India [32], and Indonesia [37]. The heightened vulnerability in rural areas is likely due to several factors that amplify the economic consequences of even minor illnesses – “rural health penalty”. Rural households often exhibit lower health literacy and sub-optimal health-seeking behaviors, coupled with higher transportation costs. These factors collectively limit access to preventive care, including vaccinations and screenings, thereby increasing susceptibility to health shocks [1, 27]. When health shocks do occur, these same factors impede access to curative services, often resulting in minor illnesses escalating to major, costly hospitalizations and surgeries [56]. Additionally, unlike urban households, most rural households in Nigeria are primarily involved in artisanal agriculture which demands consistent physical labor. Illness-related work absences directly translate to substantial income loss [3, 24–26]. Although this factor also applies to a significant proportion of urban households employed in the informal sector, these urban households are mostly involved in petty trading, where illness-related absences are more easily covered by other household members. Additionally, urban households have faster enduring recovery [21].

Before concluding, there are a few important limitations that need to be discussed. First, the cross-sectional design of our data limits our inability to control for time-varying confounders, such as the enduring effects of the COVID-19 pandemic and the insidious influence of climate change, presents a significant challenge. This is further complicated by the bidirectionality of influence: vulnerable households are more prone to health shocks due to poor living conditions and limited access to healthcare; and health shocks can impose catastrophic economic consequences on households’ consumption. Although we employed PSM to mitigate selection bias, PSM does fully address confounding introduced by these dynamic factors. Second, while this study examined the impact of any health shock on household vulnerability, it was limited in assessing the cumulative or synergistic effect of multiple exposures, or the differential impact of minor illnesses versus major illnesses such as surgeries and hospitalizations. This is a critical gap, especially given the rising burden of non-communicable diseases and the persistent impact of chronic infectious diseases like tuberculosis and HIV/AIDS on households in Nigeria, and in other LMICs more broadly. To deepen our understanding of these dynamics, future studies should prioritize collecting robust, longitudinal data with larger sample sizes that could enable the exploration of how repeated health shocks or varying degrees of health shocks, within the context of evolving disease patterns, contribute to the entrenchment of poverty.

## Supplementary information

Below is the link to the electronic supplementary material.

Supplementary Material 1

## Data Availability

The datasets generated during and/or analyzed during the current study are available in the World Bank Data Microdata Library at https://doi.org/10.48529/zd5s-tj25
