# Supplementary material for: Health shocks and households’ vulnerability to poverty in Nigeria: a quasi-experimental analysis
Source: Health Econ Rev. 2025 Jul 21;15:65. doi: 10.1186/s13561-025-00660-5 (PMC12281789; doi:10.1186/s13561-025-00660-5)
Supplement: Supplementary file 1 — Supplementary Material 1 [file 13561_2025_660_MOESM1_ESM.docx]

**Supplementary Materials**

**Table A1:** Variable descriptions, NGHS 2023/2024.

| **Variables** | **Description** |
| --- | --- |
| **Outcome variable** |  |
| Vulnerability_1 | Binary: Yes 1, No 0, if a household is vulnerable to poverty in the future based on the international poverty line of $2.15 per person per day at 2017 PPP |
| Vulnerability_2 | Binary: Yes 1, No 0, if a household is vulnerable to poverty in the future based on the international poverty line of $2.48 per person per day at 2017 PPP |
| **Independent variable** |  |
| Health shock | Binary: Yes 1, No 0, if one or more household member experienced health shock in the last 4 weeks prior to the survey |
| **Household characteristics** |  |
| Household size | Number of individuals in the household |
| Dependency ratio | The ratio of the number of household members aged less than 15 and greater than 64 to the number of household members aged 15 to 64 years |
| Sex of household head | Binary: Male 0, Female 1, sex/gender of the household head |
| Age of household head | Age of head of household (years) |
| Education of household head | Binary: 1 if education of household head is primary education or less, 0 if the education of household head is secondary or higher |
| Employment of household head | Binary: 1 if household head is unemployed, and 0 if employed |
| Household wealth quintile** | PCA summary scores for household assets categorized into five quintiles: Poorest 0, Poor 1, Middle 2, Rich 3, and Richest 4. |
| Health insurance | Binary: Yes 0, No 1, for health insurance for members of the household |
| Housing condition | Binary: Good 0, Poor 1, for the housing construction material of the house |
| **Community characteristics** |  |
| Residence | Binary: 1 for rural residents; 0 for urban residents, for the setting of the household |
| Region/Zone | Variables for geopolitical region/zone of the country: North Central 0, North East 1, North West 2, South East 3, South South 4, and South West 5. |
| State | Dummy variables for State |
| Local government area (LGA) | Dummy variables for LGA |
| Socioeconomic infrastructure index | PCA summary scores for socioeconomic infrastructure: schools, health facilities, markets, banks, and microfinance institutions available in the community. |
| Utilities infrastructure index | PCA summary scores for index that includes availability of cell phone distributors (as a proxy for the availability of mobile telecommunication), post offices, police stations, fire stations, community centres, and religious centres. |
| Transportation index | PCA summary score of presence of bus stops and paved access roads. |
| **Interactions terms** |  |
| Health shock # Household wealth interaction | Interaction term for health shock and household wealth quintile |
| Health shock # Health insurance interaction | Interaction term for health shock and if the household has health insurance |

**We used raw PCA summary scores for PSM.

**Box A2:** Lists of household assets used in computing the household wealth index*, NGHS 2023/2024

| - Furniture (sofa set) | - Generator |
| --- | --- |
| - Furniture (chairs) | - Fan |
| - Furniture (table) | - Radio |
| - Plastic chairs | - Cassette recorder |
| - Mattress | - Hi-Fi (Sound System) |
| - Bed | - Microwave |
| - Mat | - Iron |
| - Bed nets (including baby nets) | - TV Set |
| - Sewing machine | - Desktop computer |
| - Gas cooker | - Laptop computer |
| - Stove (electric) | - Tablet |
| - Stove gas (table) | - DVD Player |
| - Stove (kerosene) | - Satellite Dish |
| - Fridge | - Satellite Antenna |
| - Freezer | - Musical Instrument |
| - Air conditioner | - Smart phone |
| - Washing Machine | - Feature phone |
| - Electric Clothes Dryer | - Basic mobile phone |
| - Bicycle | - Landline telephone |
| - Motorbike | - Inverter |
| - Cars and other vehicles |  |

*We computed the household wealth index using principal component analysis (PCA) of the response (yes or no) to these household assets.

**Table A3**: Covariate balance before and after matching for rural households, NGHS 2023/2024

| **Variable** | **Unmatched/Matched** | **Exposed households** | **Control households** | **% Bias*** |
| --- | --- | --- | --- | --- |
| Household size, mean | U | 7.87 | 7.06 | 19.0 |
|  | M | 7.84 | 7.73 | 2.5 |
| Dependency ratio, mean | U | 1.36 | 1.37 | -1.1 |
|  | M | 1.36 | 1.36 | -0.2 |
| Gender of household head, % | U | 0.24 | 0.22 | 5.9 |
|  | M | 0.24 | 0.24 | 1.6 |
| Age of household head, mean | U | 54.77 | 53.33 | 9.6 |
|  | M | 54.75 | 54.40 | 2.3 |
| Education of household head, % | U | 0.21 | 0.20 | 2.9 |
|  | M | 0.21 | 0.23 | -3.3 |
| Employment of household head, % | U | 0.88 | 0.87 | 3.9 |
|  | M | 0.88 | 0.90 | -5.1 |
| Household wealth index score, mean | U | -0.74 | -0.95 | 11.9 |
|  | M | -0.75 | -0.69 | -3.4 |
| Health insurance, % | U | 0.99 | 0.99 | 11.9 |
|  | M | 0.99 | 1.00 | -2.1 |
| Housing condition, % | U | 0.50 | 0.48 | 4.7 |
|  | M | 0.50 | 0.50 | -0.3 |
| Access to safe water, % | U | 0.42 | 0.39 | 7.2 |
|  | M | 0.42 | 0.41 | 2.4 |
| Adequate sanitation, % | U | 0.44 | 0.55 | -21.9 |
|  | M | 0.44 | 0.43 | 1.7 |
| Socioeconomic infrastructure index, mean | U | -0.69 | -0.66 | -1.6 |
|  | M | -0.70 | -0.75 | 2.7 |
| Utilities infrastructure index, mean | U | -0.42 | -0.31 | -8.6 |
|  | M | -0.42 | -0.42 | 0.5 |
| Transportation infrastructure index, mean | U | -0.16 | -0.27 | 9.2 |
|  | M | -0.16 | -0.16 | 0.2 |
| **Sample** | **Mean Bias** | **Med Bias** | **Rubin’s B** | **Rubin’s R** |
| Unmatched | 8.5 | 7.9 | 43.8 | 0.84 |
| Matched | 2.0 | 2.2 | 9.1 | 1.08 |

*This column presents the standardized percentage bias, both before and after matching.

**Table A4**: Covariate balance before and after matching for urban households, NGHS 2023/2024

| **Variable** | **Unmatched/Matched** | **Exposed households** | **Control households** | **% Bias*** |
| --- | --- | --- | --- | --- |
| Household size, mean | U | 6.55 | 6.00 | 15.0 |
|  | M | 6.50 | 6.76 | -6.9 |
| Dependency ratio, mean | U | 1.14 | 1.15 | -1.3 |
|  | M | 1.14 | 1.02 | 11.6 |
| Gender of household head, % | U | 0.21 | 0.24 | -8.5 |
|  | M | 0.21 | 0.20 | 2.6 |
| Age of household head, mean | U | 53.65 | 52.89 | 5.1 |
|  | M | 53.56 | 53.01 | 3.7 |
| Education of household head, % | U | 0.23 | 0.23 | 0.5 |
|  | M | 0.23 | 0.24 | -3.5 |
| Employment of household head, % | U | 0.89 | 0.90 | -4.7 |
|  | M | 0.89 | 0.90 | -3.5 |
| Household wealth index score, mean | U | 1.40 | 1.74 | -12.2 |
|  | M | 1.42 | 1.56 | -5.3 |
| Health insurance, % | U | 0.97 | 0.95 | 9.7 |
|  | M | 0.97 | 0.96 | 7.6 |
| Own house, % | U | 0.46 | 0.52 | -11.4 |
|  | M | 0.46 | 0.45 | 2.9 |
| Housing condition, % | U | 0.81 | 0.79 | 4.4 |
|  | M | 0.81 | 0.78 | 5.4 |
| Access to safe water, % | U | 0.21 | 0.16 | 14.0 |
|  | M | 0.21 | 0.19 | 3.7 |
| Adequate sanitation, % | U | 0.21 | 0.19 | 5.4 |
|  | M | 0.21 | 0.18 | 8.1 |
| Socioeconomic infrastructure index, mean | U | 1.31 | 1.45 | -6.4 |
|  | M | 1.31 | 1.17 | 6.1 |
| Utilities infrastructure index, mean | U | 0.68 | 0.83 | -8.7 |
|  | M | 0.68 | 0.66 | 1.0 |
| Transportation infrastructure index, mean | U | 0.40 | 0.55 | -16.5 |
|  | M | 0.40 | 0.39 | 0.9 |
| **Sample** | **Mean Bias** | **Med Bias** | **Rubin’s B** | **Rubin’s R** |
| Unmatched | 8.2 | 8.5 | 31.2 | 1.19 |
| Matched | 4.9 | 3.7 | 22.7 | 1.04 |

*This column presents the standardized percentage bias, both before and after matching.

**Figure A5**: Love plot of standardized percentage bias before and after propensity score matching for rural households, NGHS 2023/2014

.
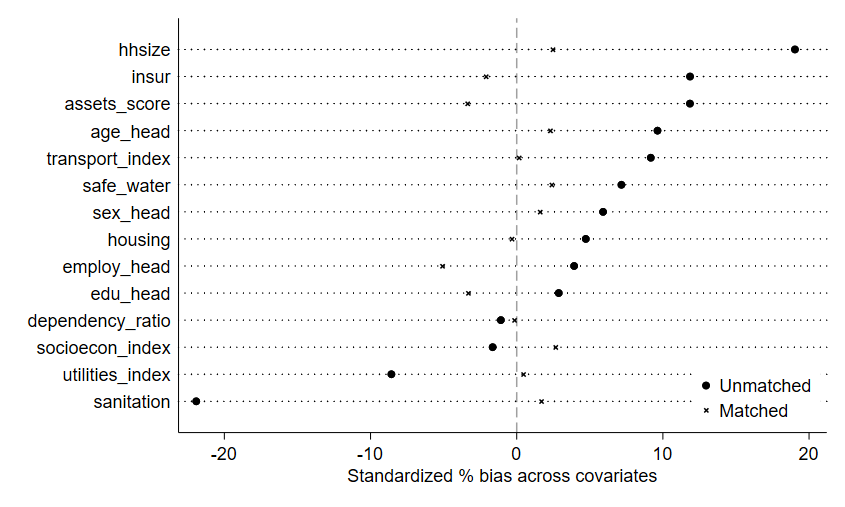


**Figure A6**: Matching graph of the propensity score before and after propensity score matching for rural households, NGHS 2023/2014.


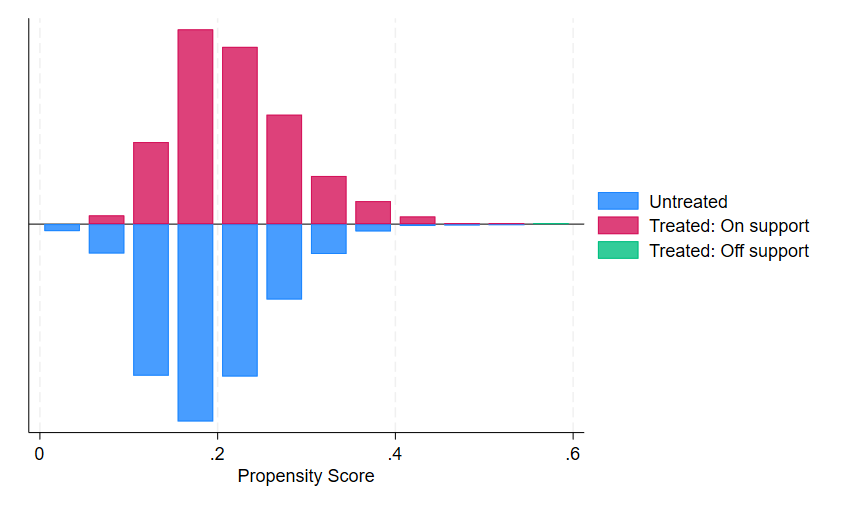


**Figure A7**: Covariate balance with propensity scores for rural households, NGHS 2023/2014.


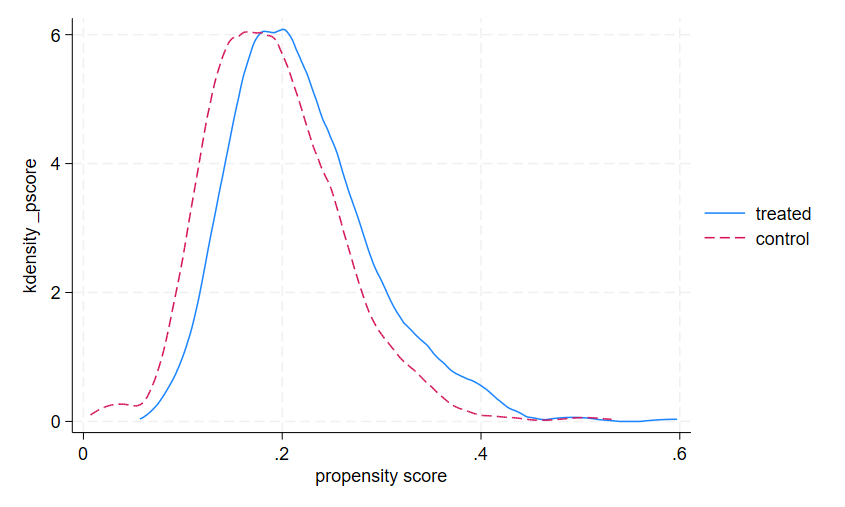


**Figure A8**: Love plot of standardized percentage bias before and after propensity score matching for urban households, NGHS 2023/2014.


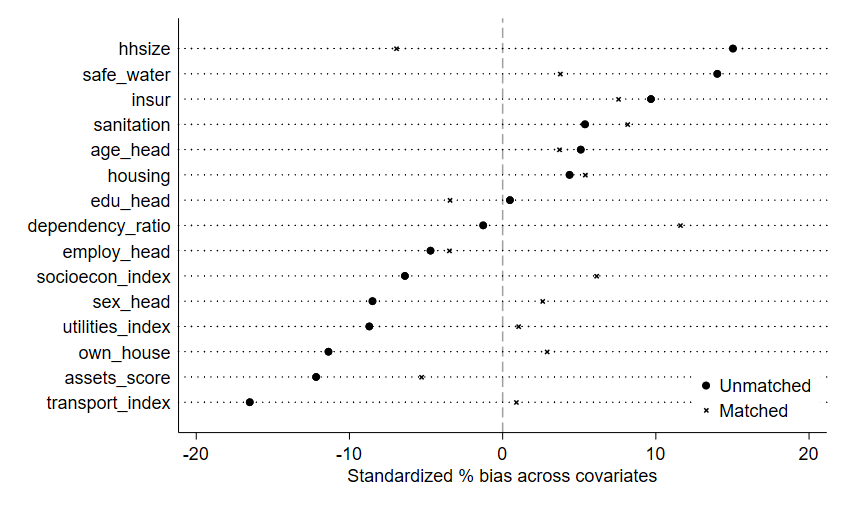


**Figure A9**: Matching graph of the propensity score before and after propensity score matching for urban households, NGHS 2023/2014.


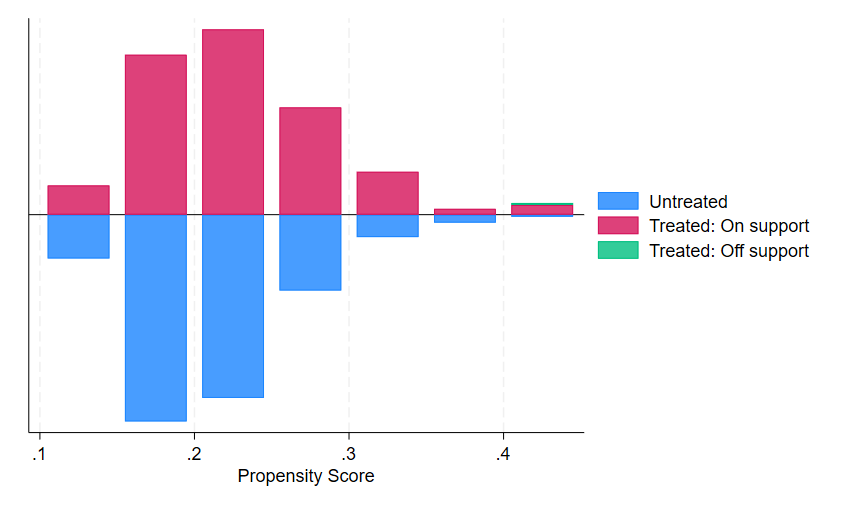


**Figure A10**: Covariate balance with propensity scores for urban households, NGHS 2023/2014.


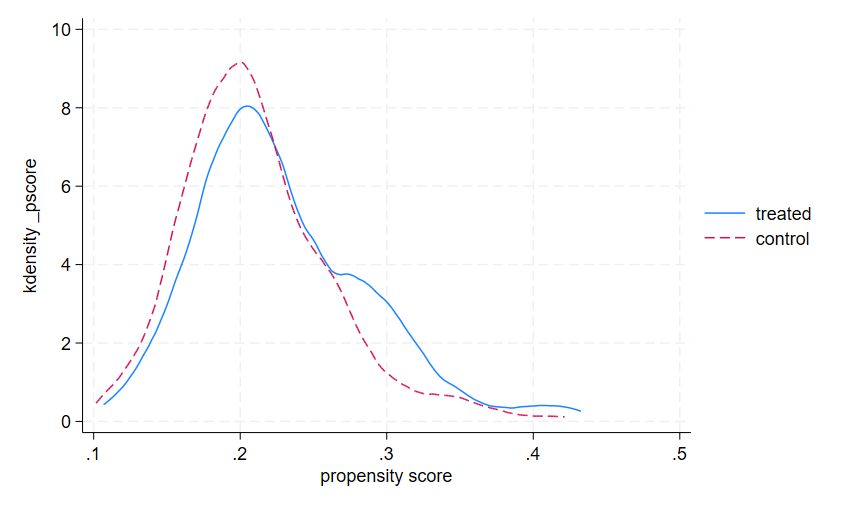


.
